# Correlation Between Microstructure and Mechanical Performance of an L-PBF 316L Alloy with an ISE-Free Parameter

**DOI:** 10.3390/ma19142932

**Published:** 2026-07-08

**Authors:** Giovanni Maizza, Ahmad Atef Abdullatef Hamed, Alberto Albanese, Maria José Marques

**Affiliations:** 1 Department of Applied Science and Technology, Politecnico di Torino, 10129 Torino, Italy; alberto.albanese@polito.it; 2 Department of Industrial Engineering, University of Rome “Tor Vergata”, 00133 Rome, Italy; 3 Department of Chemical and Biological Engineering, Faculty of Engineering, University of Porto, 4200-465 Porto, Portugal; mjvaz@fe.up.pt; 4 Department of Physics, University of Coimbra, CFisUC, 3004-516 Coimbra, Portugal

**Keywords:** laser powder bed fusion, L-PBF, performance line instrumented indentation test, PL-IIT, 316L, austenitic stainless steel, EBSD, indentation size effect, multiload/multiscale IIT

## Abstract

The optimization and the engineering development of additive manufacturing (AM) products both require accurate, non-destructive techniques to extract their mechanical performances. The Instrumented Indentation Test (IIT) has such a potential, although it currently lacks standard procedures that are suitable for analyzing materials which are affected by internal residual stress (RS). Additionally, nanoindentation testing suffers from the presence of indentation size effects (ISE), which hamper the possibility of correlating the measured mechanical performance at different indentation depths or peak loads using the standard indentation hardness (H_IT_) and modulus (E_IT_). This paper presents a novel IIT methodology that is based on new indentation parameters, namely the loading stiffness rate (LSR) and the rate-derived hardness (H_R_), which are then used to assign the desired mechanical performances of an L-PBF 316L austenitic stainless-steel alloy obtained via multiload/multiscale IIT strategy. The mean values of LSR, H_R_, H_IT_, and E_IT_ on the macroscale were 57.3 ± 1.4 GPa, 2.33 ± 0.059 GPa, 2.41 ± 0.13 GPa, and 201 ± 7.8 GPa, respectively, whereas on the nanoscale they were 56.1 ± 5.1 GPa, 2.30 ± 0.21 GPa, 3.00 ± 0.36 GPa, and 219 ± 24 GPa, respectively. Unlike the standard H_IT_, the new indentation parameters of the nano- and macro-IITs are within the standard deviation, proving their ISE-free property. The obtained E_IT_ was slightly higher than the reference Young’s modulus (~190 GPa) of the 316L stainless steel. The loading secant stiffness versus depth plot can be used to assess the susceptibility of RS to relax during indentation, which is an important performance factor for the engineering design of AM components. The successful correlation that has been found between electron backscatter diffraction (EBSD) analysis (in terms of crystal anisotropy, grain size, and dislocation density) and nanoindentation testing at three subregions of the core zone of the investigated deposit confirms the validity of the proposed methodology. The proposed methodology is a step towards the full determination of the three Ps, that is, process, properties, and performance of advanced AM products.

## 1. Introduction

Laser Powder Bed Fusion (L-PBF), which is also known as Selective Laser Melting (SLM), enables complex structural and functional metallic components to be multiform fabricated layer by layer [1]. The austenitic AISI 316L stainless steel (SS) fabricated by L-PBF is one of the most frequently investigated alloys, because of its outstanding mechanical and corrosion resistance properties, which are of great interest in many industrial sectors [2]. The mechanical properties of L-PBF 316L SS deposits have been significantly improved, compared to their conventionally processed counterparts [3]. Such improvements have been ascribed to the large dislocation density concentrated along the nanoscopic walls of sub-microscopic cellular sub-grains. The latter form colonies of similar orientation that are embedded in much larger grains, and they are confined by high angle grain boundaries (HAGBs) [4,5]. Such hierarchical and heterogeneous microstructures are determined by the high solidification/cooling rates and the steep thermal gradients that build up during the repeated remelting and re-solidification cycles of the underlying layers. The directions of the thermal gradients vary continuously. These directions drive the growth direction of the sub-grain colonies [6] within the grains during the deposition process. The final L-PBF microstructure of 316L SS is closely linked to the multiscale residual stress (RS). The latter may build up either across microscopic sub-grains (short range RS) or over the entire deposit volume (long-range RS) [7,8]. It has been shown that the RS in L-PBF 316L SS can reach several hundred MPa, as has been measured by Gel’atko et al. [9] using X-ray diffraction (XRD). As a result, the microscale and macroscale mechanical performances of the deposit depend on its microstructure (interfaces, grain boundaries, precipitates, lattice defects), multiscale RS, and crystallographic texture (if any) [10].

The mechanical properties of L-PBF 316L SS have been extensively studied in the literature using tensile tests [11,12,13,14], instrumented indentation tests (IIT) [10,15,16,17,18], and Vickers hardness [19,20,21]. Tensile tests are traditionally used to extract the bulk mechanical properties of a material from standard samples under the assumption of a uniform and homogeneous distribution of the applied stress acting over their cross-section areas. However, as the L-PBF microstructure and chemical composition [22] vary pointwise across the deposit cross-section, the measured tensile properties are no longer specific to the tested material. In addition, the tensile test properties of a sample should be considered as being ‘apparent’, since the short-range and long-range RS are closely linked with the microstructure and with the entire geometry of the sample over the elastic and plastic ranges.

On the other hand, nanoscale and macroscale IITs (n-IITs and M-IITs) can be used to sense the mechanical properties at specified local regions of an L-PBF deposit, in the form of a single imprint, along a line, or over a selected surface map. Because of the presence of multiscale RS and pointwise changes of the mechanical properties across L-PBF deposits, IIT measurements can be conducted to determine the local mechanical performance at a specified depth of a deposit, rather than its true bulk properties. Moreover, some of the embodied RS may be relaxed by the penetrating indenter, either elastically or elastoplastically, during IIT at the specified indentation depth. The RS underneath the indentation is likely to undergo elastic relaxation during IIT but be practically unaltered after IIT, and this affects the measured indentation properties to a certain extent. This is an important feature of additive manufacturing (AM) components, which should be borne in mind, especially at their design stage, as conventional engineering design principles are not fully applicable to these products since no unique material properties can be defined for them.

In a previous work [10], a dual n-IIT/M-IIT investigation was carried out extract the standard indentation modulus (E_IT_) and hardness (H_IT_) [23], in both the nanoscale (5 mN, single cycle) and the macroscale (150 N, multicycle), over two testing surfaces (X–Y and Y–Z planes) on a defect-free and chemically homogenous L-PBF 316L build (over an AISI 1020 steel substrate). The deposit was fabricated by an alternate X-scan and Y-scan with 90° rotation, and the tested sample was obtained after multiple transversal slicing electrical discharge machining (EDM) and standard metallurgical preparation. The primary goal of the paper was focused on the extraction of tensile-like ultimate tensile strength (UTS) parameter by M-IIT (=H_IT_/3, via Tabor’s approximation). The results showed a good consistency with true tensile UTS values found by several researchers; however, the other indentation properties exhibited large deviations against the literature. Detailed investigations of the data from 10-multicycle M-IIT clarified severe compliance problems between the tested sample and its holder attributed to severe in situ relaxation of remnant stresses in the samples and inherent distortions caused by its initial slicing despite the accurate calibration tests (according to ISO 14577 [23]) performed initially. This investigation highlighted the limitations of the current standard regarding the mechanical characterization of materials with significant RS, such as L-PBF materials. In addition, the properties of n-IIT are naturally affected by indentation size effects (ISE) [24], hence impeding any correlation between n-IIT and M-IIT measurements. Thus, more appropriate IIT approaches are needed to interpret IIT measurements in the case of L-PBF alloys and deposits.

For instance, an alternate model, which has been found to accurately fit the loading ICs in many materials, is Bernhardt’s law [25]. This consists of a quadratic and a linear term. The coefficient of the quadratic term has been denoted as the loading stiffness rate (LSR) [15], that is, the slope of the loading stiffness (S_h_) versus the indentation depth (h) plot. Although this stiffness is not the true tangent to a standard IC, which instead is its secant, its main advantage here is its easy and accurate computation instead of the rather complex derivatives to data points that are frequently affected by annoying noise. A useful relationship with the former is that, for general ICs described by a conventional power law fitting function, a constant scale factor (of around 2) exists between the secant and the tangent stiffnesses. Furthermore, the loading secant stiffness rate has been shown to be independent of ISE [26] and to be related to an intrinsic material property [27]. Inspired by the significant impact emerged from the investigation in [10], Maizza et al. [15] proposed a comprehensive methodology that used n-IIT, with a fixed peak load, to characterize the mechanical performance of some key regions of an L-PBF 316L deposit, which were denoted as performance zones (PZs). Nanoindentations were performed along a set of performance lines (PLs) that passed through the PZs to assess the presence of any possible anisotropic effects in the mechanical performance across the deposit. The relevant novelties introduced in this paper are as follows: (i) Bernhardt’s law for fitting the loading curve, (ii) the introduction of the loading secant stiffness vs. indentation depth plot, and (iii) the definition of new ISE-free LSR indentation parameter to complement the existing indentation parameters, H_IT_ and E_IT_. LSR was proved effective in clearly elucidating the presence of long-range RS at various indentation points affected by different RS states. Despite the positive and encouraging results demonstrated in this paper, however, the proposed methodology required further verifications and correlation with the microstructure. Moreover, the mechanical response in the central region of the deposit (the core zone, CZ) could not be determined satisfactorily, due to a mismatch of the measured indentation properties. Although the CZ was affected less by the deposit edges and the substrate, the complex tri-axial RS state existing in the CZ hampered its full characterization with just one single indentation test load.

In this study, a multiple-peak-load, multiscale IIT methodology has been designed to elucidate the mechanical behavior, across the CZ, of a geometrically similar L-PBF 316L deposit, which, however, was different in size, deposition strategy, and substrate material. The proposed IIT methodology is based on the combination of n-IIT mapping and M-IIT along two PLs. Since the latter tracks the mechanical response that is affected by the cooling edges and the substrate, the two approaches offer a mechanical bridge between the analyzed CZ and the remaining volume of the deposit. Multiple peak loads permit the RS to be probed at different depths below the indented (Y–Z) surface (scanning direction X, transversal direction Y, and building direction Z). The extraction of the standard indentation properties from the recorded data is conducted according to the ISO standard [23], and they are complemented with the LSR to determine the mechanical performance, to discriminate the RS effect across the CZ, and, ultimately, to enable their correlation with the microstructure parameters found by electron backscatter diffraction (EBSD) to be determined. The current investigation resolves the issues in [10,15] by (i) demonstrating the ISE-free property of the newly proposed LSR and H_R_ through the multiload/multiscale IIT strategy, (ii) assessing the susceptibility of RS relaxation using the S_h_-h plots, and (iii) attempting a correlation between the new indentation parameters and the microstructure parameters obtained from EBSD analysis.

Although n-IIT grid mapping of small regions has already been employed in the literature [28], its application to a relatively larger region, as in the current study, has been aimed at determining the performance of a deposit on microscopic regions in order to enable a correlation to be made between the nanoscale and macroscale mechanical performances.

## 2. Materials and Methods

### 2.1. L-PBF 316L Deposit and Sample Preparation 

Pre-alloyed Renishaw 316L-0407 powder (Renishaw Iberica, Barcelona, Spain) was used to fabricate the investigated L-PBF deposit (120 mm long in the X direction, 17 mm wide in the Y direction, and 17 mm high in the Z direction) over a mild steel substrate (135 × 31 × 26 mm^3^). The chemical composition of the deposit, analyzed by the G.N.R. S7 Metal Lab Plus (G.N.R., Agrate Conturbia, Italy) optical emission spectrometer, is reported in Table 1. The L-PBF parameters are listed in Table 2 according to the data provided by the manufacturer. An island scanning strategy was used, with 67° rotation of the laser scanning direction after each layer.

**Figure 1:**
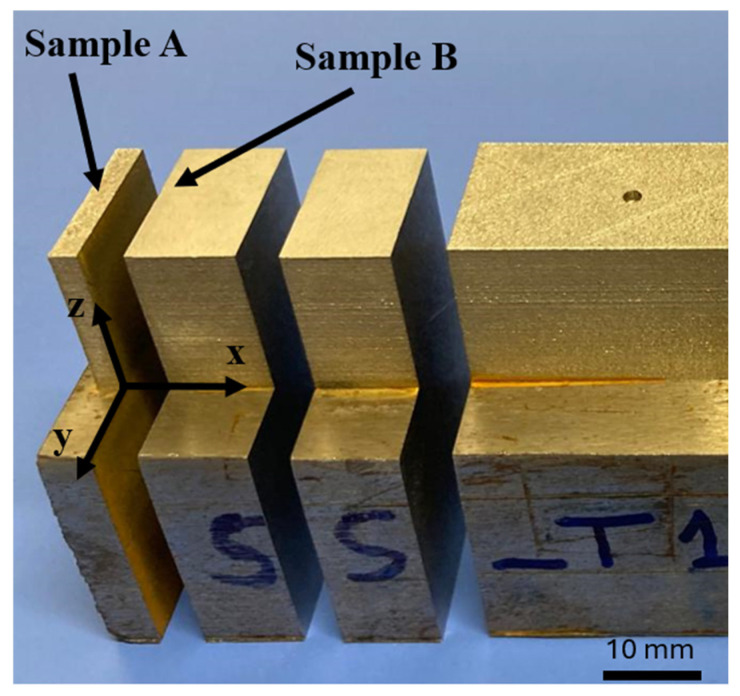
Sliced samples for the metallographic analysis (Sample A) and indentation tests (Sample B).

Two adjacent samples, denoted as samples A and B, were sliced transversally from the original deposit by means of EDM (Figure 1), for the metallographic examination and indentation testing, respectively. Sample A coincided with one end of the entire deposit, while sample B shared its mirror Y–Z cut face with the former. The coinciding surfaces of samples A and B were ground with SiC smearing paper (up to 4000 grits), and finally polished using 6-, 3-, and 1-μm diamond particle suspensions.

### 2.2. Multiple Peak Load Nano-/Macro-Indentation Strategy

A square region (5.5 × 5.5 mm^2^) of the deposit core (CZ) of sample B was subjected to 576 equally spaced (200 μm) nanoindentations (Anton Paar Hit 300, Graz, Austria), under load control, using a modified Berkovich indenter. Since a multi-load strategy had been designed (50, 100, 150, and 200 mN), the whole probed region was divided into sixteen cells. Each cell contained four sub-cells. Each sub-cell was filled with an equally spaced (100 μm) 3 × 3 n-IITs matrix under one of the four peak loads. The cells were equally (600 μm) spaced with one another. The nanoindentations were carried out under a constant loading/unloading rate (120 mN/min) and holding time (10 s). The n-IITs were executed before the M-IITs to prevent compliance problems caused by possible distortions of the sample upon the consequent relaxation of the long-range RS [10].

**Figure 2:**
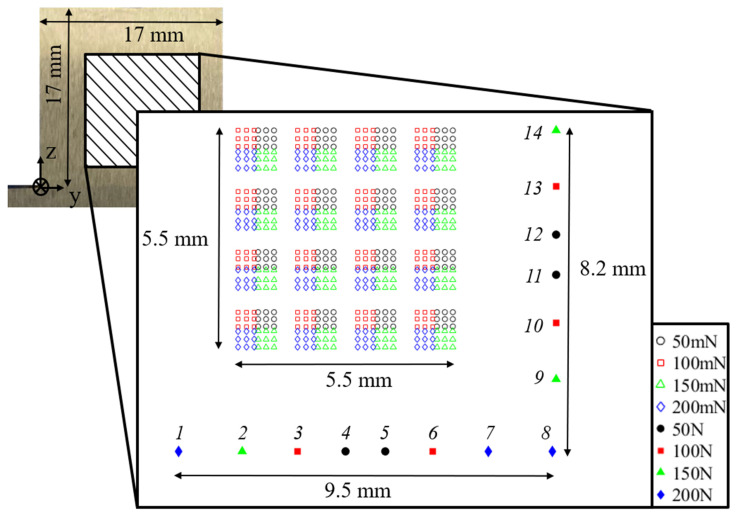
Sketch of the designed nano-/macro-indentation strategy.

Subsequently, a series of 14 M-IITs (Zwick Roell ZHU 2.5, Vickers indenter, Ulm, Germany) were performed along two PLs, as follows: horizontally below the CZ (8 indents) and vertically, on its right-hand side (6 indents) at 3 mm from the substrate and from the right border, respectively. Only two lines were selected, for deposit symmetry reasons, and to mitigate any possible distortion resulting from RS relaxation during M-IIT, as it typically interacts with long-range RS. The selected peak loads were 50, 100, 150, and 200 N, under a constant holding time at the peak load (30 s), and constant loading and unloading rates (1 and 4 N/s, respectively). Four M-IIT cycles were repeated for each indentation using the same peak load to obtain a more accurate measurement of E_IT_ [30]. The presented and discussed H_IT_ and E_IT_ values are the ones that were obtained after the fourth cycle. The two M-IIT PLs were sufficient to bridge the mechanical response trend on the macro-scale generated from the outer portion of the deposit to the internal CZ along its horizontal (bottom) and vertical (right-side) boundaries. We used multiple peak indentation loads to prove the negligible influence of the load on the LSR. The loads for the n-IITs and M-IITs were suggested by other works [10,15,16,17,18,19] for comparison. A sketch of the overall designed multiload nano-/macro-indentation strategy is depicted in Figure 2.

### 2.3. Bernhardt’s Indentation Law

Benhardt [25], and later Fröhlich et al. [31], proposed a correction of Kick’s quadratic law [32] to improve the fitting of the loading curve with experimental data in the case of sharp indenters, as follows:

P = LSR · h^2^ + b · h, (1)

where P and h are the indentation load and penetration depth, respectively, whereas LSR and b are fitting constants. However, there is no consensus on the physical meaning of either of the fitting parameters. The LSR was considered to be related to the true hardness of the material [33], the load-independent (ISE-free) hardness [34], the plastic work done to produce the permanent volume deformation [31,35], and a factor that discerns the plastic deformation modes [36]. On the other hand, b was attributed to ISE [34], surface stresses [26], the work done to create new surfaces [35], and the indenter’s bluntness [36].

Alternatively, Bernhardt’s law can be written in terms of the secant stiffness, S_h_, which is defined as follows:

S_h_ = LSR · h + b. (2)

The S_h_-h curves of many metallic materials, such as the current L-PBF 316L SS, can be described by a straight line, except for the small depth interval near the origin. In such a depth interval, the secant stiffness curve may deviate from linearity, because of several factors (e.g., ISE, indenter bluntness, and other surface-related anomalies). S_h_-h curves are particularly useful to explain the elastoplastic material response during IIT in an alternate manner, including any possible stress relaxation events. Thus, they can be used to track the susceptibility of an L-PBF material to RS relaxation upon indentation, which is an important engineering design index.

Unlike the conventional indentation hardness, H_IT_, the LSR parameter exhibits a unique ISE-free feature and, thus, can numerically be converted into a conventional hardness index, via the geometrical factor, g (to account for the shape of the indenter, i.e., 24.5 and 24.49 for the Vickers and modified Berkovich indenters, respectively) [23], namely through the following relationship:

H_R_ = LSR/g, (3)

It should be noted that H_IT_ is, by definition, function of the contact depth, h_c_, which, in turn, is calculated using the standard maximum penetration depth [23]. The latter includes both the loading and holding displacement effects. For clarity, we denote it here as h_2max_. Conversely, H_R_ only depends on the maximum loading penetration depth, denoted here as h_1max_. Here, the use of geometrical factor g presupposes an ideal contact condition, which is believed to be more likely during loading than during holding, where the material relaxes the loading stresses. Consequently, H_R_ is more reliable than H_IT_ because it (i) is ISE-free, thus multiple load indentation tests can be correlated, (ii) properly reproduces the ideal geometry of the indenter upon loading, (iii) is free from any assumptions for calculating h_c_, and (iv) is based on a direct measured quantity, h_1max_. Thus, H_R_ should be viewed as a reliable intrinsic material indentation property, as the S_h_-h curve is accurately described by a straight line, e.g., a constant LSR, from the (sub)surface to the bulk (up to h_1max_). The use of H_R_ is also advantageous when it is necessary to compare the response of a material at different penetration depths (or peak loads), which is particularly convenient in parts or deposits affected by RS. As the latter circumstance is not covered by the current ISO 14577-2015 standard, it is the subject of the present work.

In this paper, ICs, derived from n-IIT and M-IIT, were fitted with Bernhardt’s law. H_R_ was used to complement the standard indentation properties for the determination of the mechanical performance of an L-PBF 316L deposit under different load conditions and in the presence of RS effects.

### 2.4. Metallographic Analysis

First, 10 optical micrographs were taken, using optical microscopy (DMI 3000 M, Leica, Wetzlar, Germany), and then analyzed using ImageJ (1.54g) software [37] to assess the average porosity level of the given deposit. Subsequently, the sample A was etched for 10 s with aqua regia for inspection by optical and scanning electron microscopy (SEM-FEI M-EDX Sirion 400 NC, Thermo Fisher Scientific, Hillsboro, OR, USA). The EBSD analysis (TESCANS 9000G, Tescan group, Brno, Czech Republic) was carried out at a 1.5 k× to 12 k× magnification and 20 kV at a scanning step size of 1 μm, after standard polishing procedure. Three prominent 500 × 500 μm^2^ subregions were selected for further EBSD analysis, on the basis of the n-IIT results.

## 3. Results

### 3.1. Microstructure

**Figure 3:**
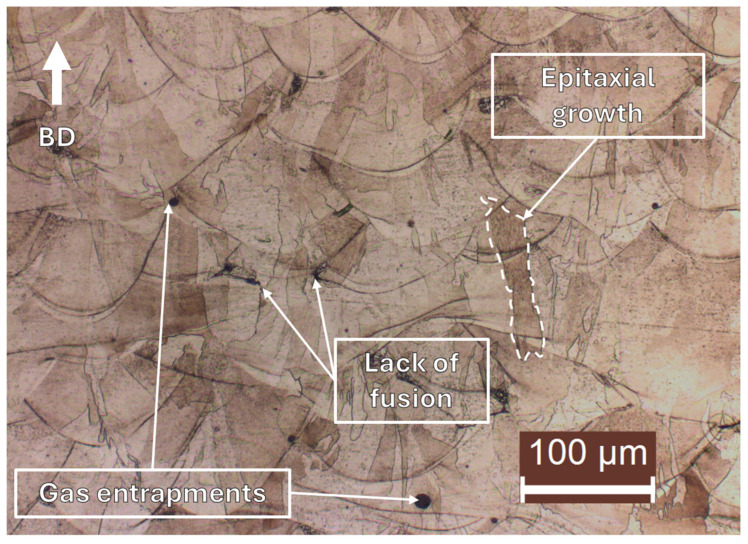
Optical micrograph of the etched L-PBF 316L microstructure.

The density of the deposit, estimated using OM image analysis, was 99.4 ± 0.4%. Figure 3 shows an OM image of the microstructure after etching, where a few typical L-PBF defects, such as lack of fusion and gas entrapment pores, can be seen. Figure 3 displays a typical microstructure, resulting from a 67°-rotation scanning strategy per layer [38], although the melt pools do not clearly define the individual layers. Nevertheless, some epitaxial growth can be observed along the building direction (BD, the vertical direction in Figure 3).

**Figure 4:**
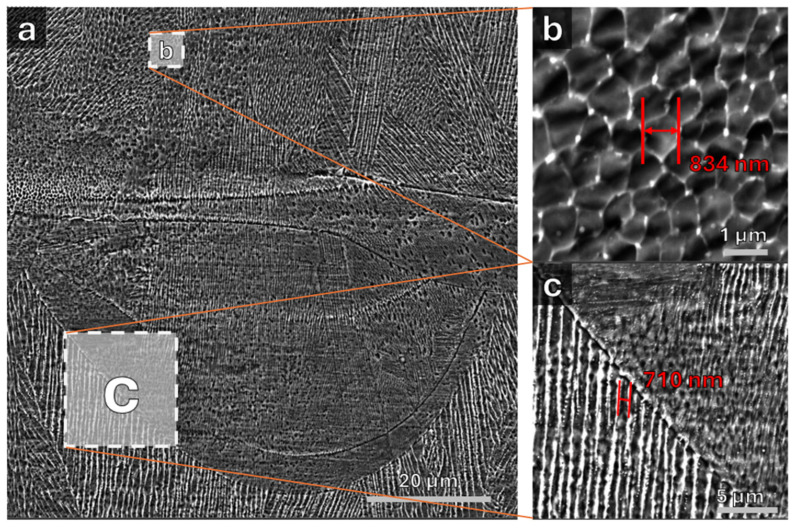
SEM images of the microstructure in (**a**) the center of the Y–Z plane. Insets (**b**,**c**) show magnifications of the cellular and columnar substructures, respectively, and their characteristic sizes.

The SEM images of the microstructure at the center of the CZ (Figure 4) show a resolved cellular substructure made up of longitudinal dendritic cells (Figure 4c) oriented along different angles, together with their hexagonal cross-sections (Figure 4b), both of which are embedded into colonies which compose larger grains bound by HAGBs. The average size of such dendritic cells (Figure 4a), as measured across several SEM images in different zones of the melt pool, is 651 ± 194 nm.

### 3.2. n-IIT Grid Map Results

Table 3 shows the mean values of the four relevant n-IIT properties obtained from the probed grid along with the relative standard deviation (RSD = standard deviation/mean value) for each load from the investigated CZ. For convenience, the last line in Table 3 summarizes the mean values of the indentation properties and the corresponding RSD percentage after averaging over all the loads. The estimated load-averaged mean values of LSR, H_R_, H_IT_, and E_IT_ amount to 56.1 ± 5.1 GPa, 2.30 ± 0.21 GPa, 3.00 ± 0.36 GPa, and 219 ± 24 GPa, respectively. The table clearly underlines the influence of ISE on the indentation parameters, which is more appreciable for the conventional H_IT_ and E_IT_ indentation properties than for H_R_ or LSR, due to the lower RSD across all the loads. It can be seen that LSR, H_R_, and H_IT_ decrease for increasing peak loads, while E_IT_ increases. Conversely, the calculated RSD of all the parameters is higher at 50 mN. The b parameter for the n-IITs is always positive for all the loads, except for few n-IITs with quite irregular ICs.

**Figure 5:**
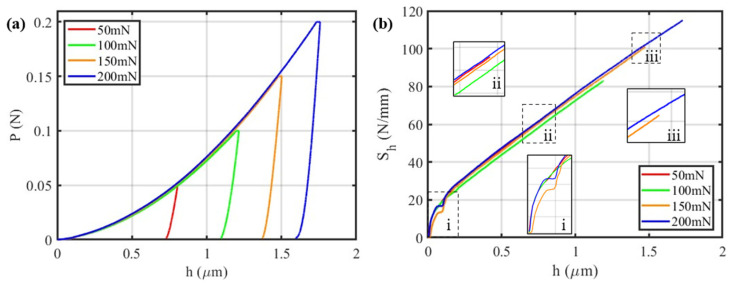
Typical n-IIT for all the peak loads: (**a**) conventional indentation curves; (**b**) secant stiffness curves.

Figure 5 shows the typical indentation curves (ICs, P-h) over the nanoscale range for different peak loads (Figure 5a) along with the derived secant stiffness (S_h_-h) curves (Figure 5b). Despite the apparent regular shape of all the ICs, the S_h_-h curves highlight their non-linear behavior near the origin (see inset i). Indeed, a “plateau” can be observed in that region for the two larger load curves (150 and 200 mN) but also observed for other indentations at lower loads, thus indicating a balance between the imposed S_h_ and the intrinsic material stiffness across the sub-surface layer. The secant stiffness curves become linear at greater depths, and their slope may eventually change locally or globally, depending on the susceptibility (or stability) of the internal RS of the material to relaxation at different depths. In inset ii, which shows the final stage of the 50-mN indentation curve (red), all the stiffness loading curves exhibit the same slope. Conversely, in inset iii, the slope of the 200 mN curve (blue) tends to decrease.

**Figure 6:**
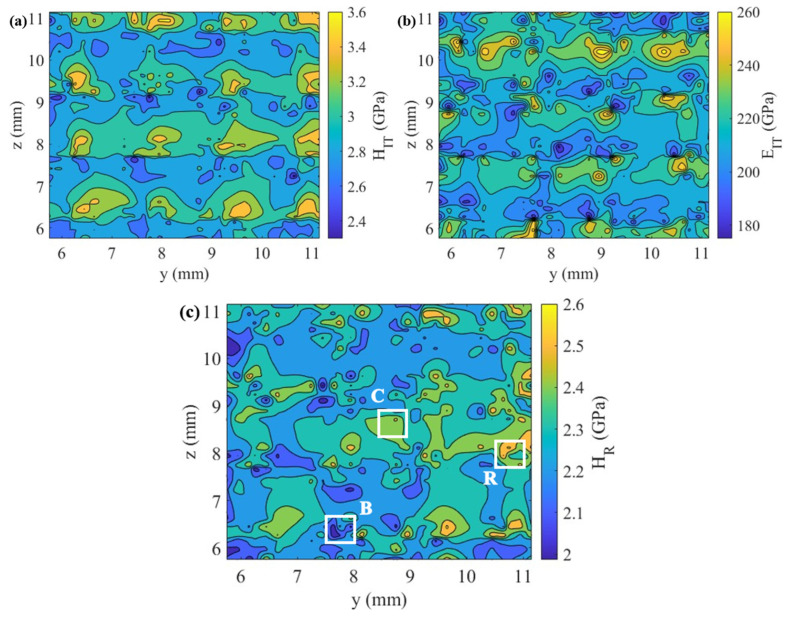
Contour plots of the indentation properties: (**a**) H_IT_; (**b**) E_IT_; (**c**) H_R_ with the three subregions selected for further EBSD analysis.

The contour maps in Figure 6 show a two-dimensional variation in the three indentation properties (H_IT_, E_IT_, and H_R_, respectively) across the probed grid of the CZ of the deposit. The H_IT_ plot (Figure 6a) displays an obvious periodicity, which reflects its natural dependence on the indentation load, hence underlining its high sensitivity to ISE. Identical periodicity can be observed in the E_IT_ contour map (Figure 6b), although it is of an inverse nature, because of its strong dependence on the indentation load. On the other hand, the lower sensitivity of LSR and H_R_ to ISE clearly leads to the presence of structural anisotropy in their contour plots (Figure 6c), which are located on the right side of the CZ, thus revealing an enhanced mechanical performance of this region compared to the opposite (left) side of the CZ.

Three subregions (Figure 6c), namely R, C, and B, were selected for further investigation, using EBSD, to better elucidate the microstructural factors that could lead to such differences in the mechanical performance of the CZ.

### 3.3. M-IIT PLs Results

The M-IITs results and their RSD for each peak load (50, 100, 150, 200 N) along the horizontal and vertical PLs are shown in Table 4. The mean values, averaged over all the peak loads, amount to 57.3 ± 1.4 GPa, 2.33 ± 0.059 GPa, 2.41 ± 0.13 GPa, and 201 ± 7.8 GPa, for LSR, H_R_, H_IT_, and E_IT,_ respectively. As expected, the M-IIT properties show a negligible ISE effect. The RSD values of the LSR and H_R_, upon averaging over all the loads, are lower than those of H_IT_ and E_IT_. The mean values of LSR and H_R_ at the macroscale (Table 4) compare well with their counterparts in the nanoscale (Table 3). This result supports our ambitious goal of tracking RS behavior from the nanoscale to the macroscale at different depths, while obviating any load dependence. Moreover, the macroscale-averaged (over all the loads) indentation moduli (Table 4) were slightly higher than the reference Young’s modulus (~190 GPa) of the 316L SS [39].

**Figure 7:**
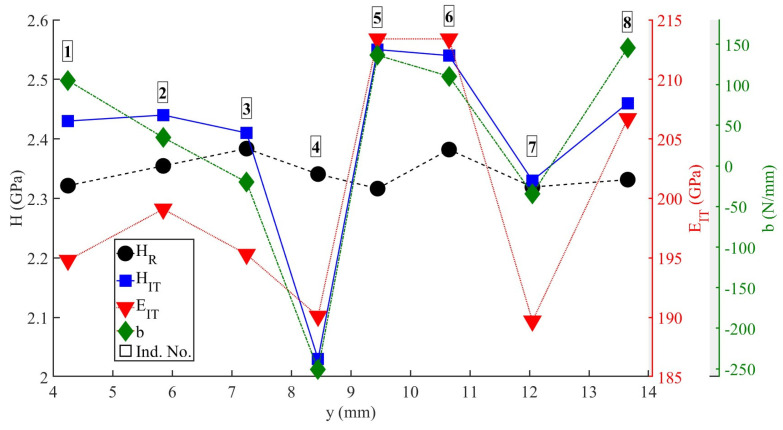
M-IIT properties along the horizontal PL. The numbers in square boxes refer to the numbers of each indentation.

**Figure 8:**
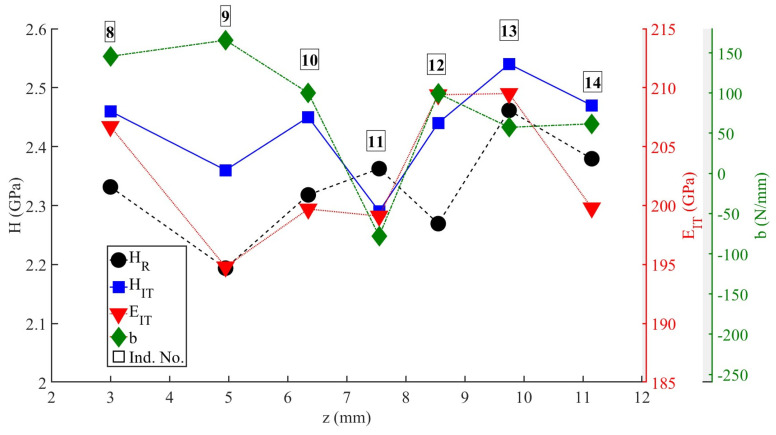
M-IIT properties along the vertical PL. The numbers in square boxes refer to the numbers of each indentation.

Figure 7 and Figure 8 illustrate the variation in the indentation properties along the horizontal (Y direction) and vertical (Z direction) PLs, respectively, which show an oscillatory trend in both directions. Any oscillations in the M-IIT indentation properties should be associated with the temperature gradients across the deposit and, hence, to long-range RS. The small oscillation amplitude of H_R_ along the horizontal PL reflects the uniform heat extraction effect, along the Y direction, exerted by the substrate from the upper L-PBF deposit. Such a horizontal oscillation amplitude is lower than that displayed along the vertical PL. H_R_ tends to increase along the vertical PL as the distance from the substrate increases, because of the lower cooling effect imparted by the substrate than on the top edge as the deposit height increases. The limit values of H_R_ (2.46 GPa being the highest and 2.19 GPa the lowest) along the vertical line were obtained for indent numbers 13 and 9, with 100 and 150 N peak loads, respectively. Indentation 13 is located near the top right edge of the CZ (Figure 2), the region that was observed to have the highest n-IITs mechanical performance. On the other hand, macroscale H_IT_ and E_IT_ suffer from much larger deviations along both the horizontal and vertical PLs.

**Figure 9:**
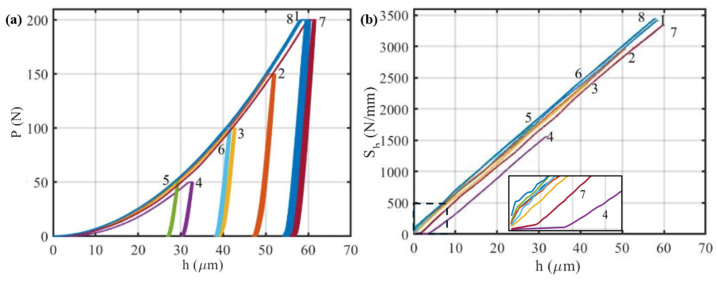
(**a**) ICs, and (**b**) secant stiffness curves for the M-IIT of the horizontal PL. The curves are labeled with the indentation numbers at the end of the loading segment of each curve.

**Figure 10:**
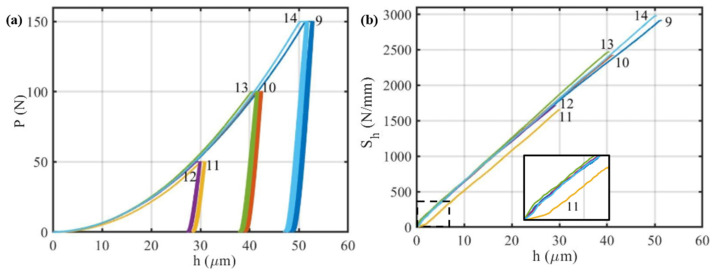
(**a**) ICs, and (**b**) secant stiffness curves for the M-IIT of the vertical PL. The curves are labeled with the indentation numbers at the end of the loading segment of each curve.

Figure 9 and Figure 10 show the M-IIT ICs along the horizontal and vertical PLs (Figure 9a and Figure 10a) and the respective secant stiffness curves (Figure 9b and Figure 10b). The ICs show typical regular profiles for metallic materials. Conversely, some S_h_-h curves (e.g., indentations 4, 7, and 11) reveal a certain degree of anomaly near the origin (see the insets in Figure 9b and Figure 10b). Such abnormal stiffness curves differ from the regular ones in that all of them experience very low stiffness while undergoing an exceptionally large displacement (a few micrometers) and thus indicate negative values of parameter b (Figure 7 and Figure 8). Parameter b is also negative for indentation 3, but it is the least negative among all indentations, and the stiffness curve is normal. However, the other curves start with a larger secant stiffness, which gradually decreases after a few micrometers in depth, and gradually approaches a steady state slope (i.e., the LSR), which has been attributed to the relaxation of the more compressive superficial RS. Moreover, all the curves tend to show an unaffected, similar LSR, regardless of the initial abnormal indentation stage in the S_h_-h plot. However, this anomaly manifests itself as a shift to higher indentation depths (i.e., increasing abnormally h_2max_) and a consequent decrease in the conventional indentation properties (H_IT_ and E_IT_). The common factor of these abnormal points (4, 7, and 11) is that they are internal points in their respective PLs. Indentations with larger LSR (e.g., indentations 13) may indicate the influence of a more compressive RS state, while those with a smaller LSR (e.g., indentation 9) may indicate that of a more tensile RS state.

### 3.4. EBSD Results

**Figure 11:**
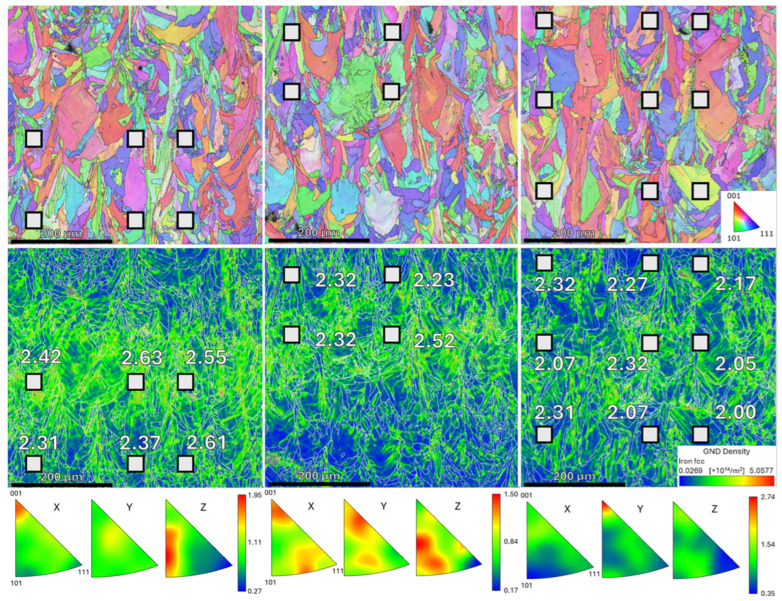
IPF maps (**top**), GND density maps (**middle**), and IPF plots (**bottom**) of the three analyzed subregions R (left), C (center), and B (right). The colors in the IPF maps correspond to crystallographic directions parallel to the building direction (Z). The white squares in the figures indicate the positions at which the local nanoindentation H_R_ values (in GPa) were measured in the three subregions.

Figure 11 shows the EBSD orientation maps (top row) with the <uvw> directions parallel to the building direction of the sample (Z), the geometrically necessary dislocation (GND) density maps (middle row), and the inverse pole figures for the three selected subregions: R, C, and B, corresponding to the columns on the left, in the middle, and on the right of the figure, respectively. The positions of the n-IITs are also shown in the IPF and GND density maps, and the H_R_ values of each n-IIT in the three subregions have been added to the GND density maps. The averaged H_R_ values of the n-IITs in each subregion were 2.48 ± 0.13, 2.35 ± 0.12, and 2.18 ± 0.13 GPa, whereas the average grain sizes measured from the EBSD analysis, expressed in terms of cross section areas, were 215, 257, and 267 µm^2^, respectively. The measured fractions of the low angle grain boundaries (LAGBs < 10°) in the three subregions were 37.8%, 37.3%, and 36.8%, respectively. No other phases, other than austenite, were detected across the three subregions. Subregion B showed a relatively stronger texture (<001> parallel to the sample transversal direction Y), while subregion C showed the weakest and broadest texture, and subregion R exhibited an intermediate texture.

**Figure 12:**
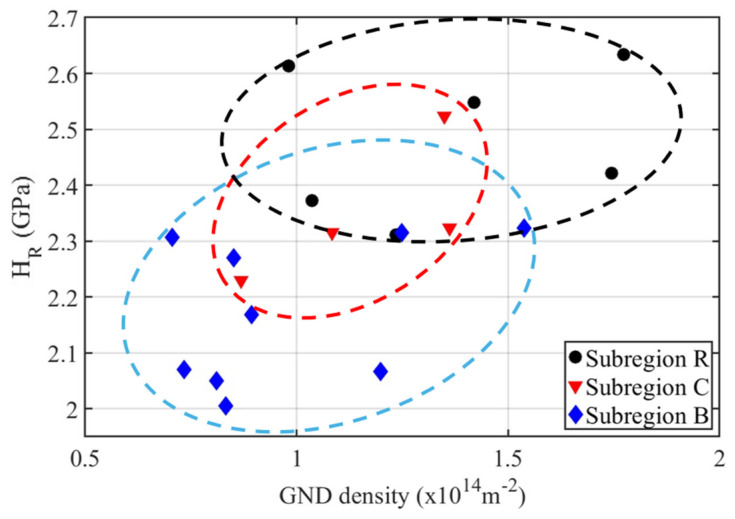
The H_R_ values of each n-IIT plotted against the average GND density for the three analyzed subregions at the same n-IIT position.

The GND density was determined from the local orientation gradients [40], using the kernel average misorientation (KAM) as a function of the intragrain lattice curvature. In this study, GND density has been used as a relative indicator of the total dislocation density that originated from the rapid solidification and fast cooling during the L-PBF process. The measured GND density peak in the three subregions was slightly more than 5 × 10^14^ m^−2^, whereas its average value was 1.14 × 10^14^ m^−2^, which compares well with the literature value for L-PBF 316L alloy [6]. The average values of the GND density at subregions R, C, and B were 1.26 ± 0.65, 1.08 ± 0.64, and 1.05 ± 0.61 × 10^14^ m^−2^, respectively. Figure 12 displays the values of the nanoscale H_R_ against the GND density values averaged over a 40 × 40 µm^2^ square region. The center of each square coincides with the indentation position. Hence, higher H_R_ values are correlated with higher GND densities, and vice versa.

## 4. Discussion

### 4.1. Secant Stiffness Curves

It is well known that the presence of RS in as-built L-PBF deposits (especially whenever the substrate is also included) affects all the stages of the indentation process, that is, during loading, holding, and unloading. However, this effect is of minor entity for n-IITs, and it is even lower for lower peak loads. On the other hand, M-IIT can dramatically alter the existing RS state and cause marked distortions of the sample, which are more severe for larger peak loads [10]. In this paper, we have attempted to circumvent this problem by carrying out M-IITs after n-IITs. The results show that standard properties H_IT_ and E_IT_ are both very sensitive to RS in the L-PBF samples, although the latter appears to be much more sensitive. In the examined L-PBF 316L alloy samples, both properties exhibited opposite trends, thereby failing to give a definite mechanical response. We introduced the LSR and the S_h_-h curves as auxiliary tools to shed more light on such ambiguous situations. Their benefit is clear, as both tools are derived from the loading curve, where contact between the indenter and the material is theoretically likely to be accounted for, even when RS is present. Hence, if any eventual unstable RS causes deviations from the regular elastoplastic response of the material on loading, these deviations can be accurately tracked by means of the S_h_-h curve by considering the changes in its slope. In the absence of RS, LSR should be proportional to Young’s modulus and to the work-hardening rate of the material (or the plastic or tangent moduli). A constant slope in the S_h_-h curves is indicative of a well-defined deformation regime, which may include an RS (elastic and/or inelastic) contribution. During the last loading stage of indentation, the L-PBF 316L SS alloy is expected to deform elastoplastically under the concurrent presence of an RS field. Any plasticity induced by the edges and tip of the indenter upon penetration will determine a partial or total RS relaxation. Its inelastic portion results in a change in LSR upon loading, whereas the elastic portion will be recovered elastically upon unloading. The S_h_-h curves also account for the initial deformation (due to indenter bluntness) and for any eventual RS acting on the surface, as well as other anomalies, such as the presence of defects (pores, carbides, oxides, and the like). The measured LSR accounts for such surface or subsurface phenomena with relatively small slope changes and essentially returns the true bulk stiffness property of the material. This further insight into the initial behavior of the material during indentation, as revealed by the S_h_-h curves, is not detectable in the original ICs. Finally, LSR includes microstructure stiffness and the stiffness exerted by stable (i.e., remnant) RS. The former stiffness, in turn, embodies two contributions, namely, material strain hardening (i.e., linked to its hardness) and intrinsic material stiffness (i.e., Young’s modulus).

The initial segment of the S_h_-h curves of the n-IITs is normally non-linear and, in some cases, it may culminate in a stiffness plateau (see inset i of Figure 5b), followed by a sudden rise in stiffness while approaching the characteristic LSR of the material. Although the initial stage is in general believed to be purely elastic, the action exerted by a blunt indenter over local accidental microstructure heterogeneities (e.g., brittle oxides or local RS), may explain the observed abnormal crushing-like phenomena near the origin of the S_h_-h curves. The indentation properties for relatively low indentation loads (or penetration depths) are more sensitive to superficial anomalies and, thus, to stiffness variations, which explains why all the n-IIT properties at 50 mN showed the largest RSD.

Conversely, at first glance, the initial elastic deformation near the origin of the M-IIT S_h_-h curves does not seem to be as appreciable as in the n-IITs. However, a deeper inspection of the M-IIT S_h_-h curves has revealed the effect of superficial anomalies, as can be seen in the insets of Figure 9b and Figure 10b for M-IITs 4, 7, and 11. These indentations start with a much lower slope than the other indentations at a few micrometers below the surface, likely due to a tensile RS state, which permits the indenter to penetrate with a minimal opposing resistance. At greater depths, the slope returns to the typical LSR values of the (L-PBF 316L) macroscopic, elastoplastic, bulk material behavior, which, to a certain extent, also includes an RS contribution. This causes a shift to the right of both the ICs and the S_h_-h curves of these indentations, which translates into abnormally larger h_2max_, and thus h_c_, values. According to [23], this shift results in lower H_IT_ and E_IT_ (Figure 7 and Figure 8). Conversely, the LSR and H_R_ values reveal somewhat fair bulk elastoplastic properties, regardless of the presence of anomalies. Such a unique feature of LSR can be explained by considering its natural decoupling from the local S_h_, which remains the only parameter that is sensitive to superficial anomalies. Accordingly, the latter may increase or decrease as a function of the strength of the anomalies, whereas LSR practically remains constant. Abnormal superficial features can be conveniently detected by observing the negative values of b of Bernhardt’s law (the y-intercept of the S_h_-h curves). The more negative b is, the more detrimental the anomaly present on the sample surface. The effect of the superficial state on this parameter has been reported in [34]. However, the distinctive negative sign of b appears to be more striking at higher peak loads, such as those in M-IIT, whereas it is less meaningful for those in n-IIT.

Apart from the origin of the S_h_-h curves, reductions in the slope during indentation suggest possible stress relaxation events caused by plastic deformation induced by the edges of the indenter. Alternatively, the slope may change if the original RS state is altered during indentation. The slope change observed during n-IITs (blue curve in inset iii of Figure 5b) involves a slight decrease in LSR, accompanied by a partial relaxation of RS. An increase in S_h_ (blue curve) under concurrent slope change shall be interpreted as an incipient strain hardening, after relaxation of RS exerted by the indenter.

**Figure 13:**
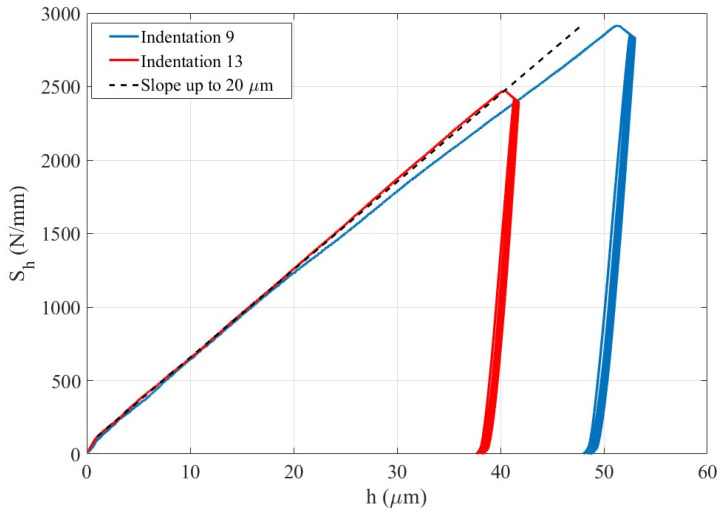
Comparison of the S_h_-h curves of M-IIT 9 (150 N) and M-IIT 13 (100 N), which share a common loading stiffness slope from the origin up to 20 µm (dotted line), but a gradual deviation of the slope of M-IIT 9 is observed with increasing depth due to a partial relaxation of a likely compressive original RS.

In general cases, a change in S_h_ or slope in the S_h_-h plot may also result from changes in crystal orientation (due to elastic stiffness), accidental presence of phases (either hard or soft), pores/cracks, or RS. Detailed inspection by means of SEM and optical microscopy at indentation points excluded the presence of hard/soft phases, pore or crack, as expected by the microstructure homogeneity, ductility, and stainless properties of the L-PBF 316L steel. Crystal orientation effect, however, could not be excluded a priori based on the results of Figure 11. Experimental observations suggested that microstructure composition, crystal orientation effect, and short-range RS effect could be combined all together to define the intrinsic properties of the material, since all of them contributed to synchronous indentation properties (i.e., all increase or decrease). Moreover, short-range RS is less sensitive to sample slicing (in locations away from the edges). This is opposite to long-range RS which may markedly alter their original self-balanced 3D state upon sample slicing or during indentation testing, especially under large indentation loads. Tensile long-range RS are more prone to relax during indentation testing. Their variation can be detected along each performance line by the presence of an inflection point in the S_h_-h plot. Conversely, a compression initial RS state may cause a comparatively smaller stress relaxation effect than a tensile initial RS. Figure 13 shows such an example. The compression state at indentation 9 has been confirmed by (superficial) X-ray diffraction (XRD) measurements. Figure 13 compares the S_h_-h curves of M-IITs 9 and 13 at peak loads of 150 N and 100 N, respectively. Both indentations share a nearly identical slope from the origin up to approximately 20 µm. With increasing load, M-IIT 9 undergoes a partial stress relaxation, whereas M-IIT 13 remains practically unaltered. This behavior has been associated in general with a rather stable initial compression RS state under indentation with the Vickers indenter. As a result, M-IIT 9 retains a less compressive RS state than its original RS state.

Moreover, the intimate link of RS with the microstructure clearly makes the separation of the two contributions by means of IIT very difficult. This topic has been a frustrating, unsolved issue for several decades and perhaps a misleading goal from at least the engineering and fabrication viewpoints. Indeed, a worthwhile and challenging task is to prevent tensile RS or to promote superficial compression RS in fatigued parts. The use of H_R_ and b parameters can help solve this issue.

**Figure 14:**
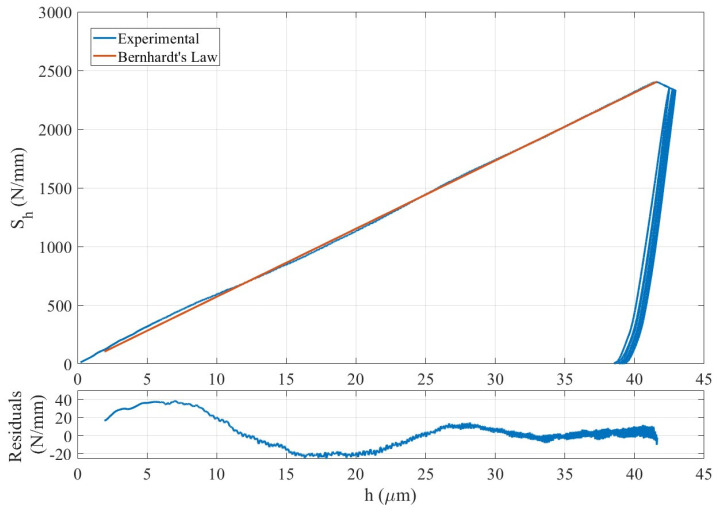
The S_h_-h curve (**top**) of Indentation 3, and the curve of Bernhardt’s law fitting residuals (**bottom**).

Periodic oscillations of S_h_ vs. h are shown in Figure 14, in terms of the fitting residuals given by Bernhardt’s law (Residuals = Experimental − Fitting) in the case of indentation 3. The period of the oscillations has been estimated as 20 ± 5 µm, which corresponds to approximately 200 µm of the deformed material under the indenter (assuming the deformed depth is 10× the penetration depth) in the longitudinal (X) direction. Incidentally, in the investigated L-PBF 316L alloy, this is close to twice the hatching distance used during laser fabrication. Similar periodic oscillations of the RS in L-PBF 316L were observed [41] along the building direction when using XRD analysis. The number of peaks and the period length provide a stability index of the characteristic RS induced by the L-PBF process. The poor stability of RS on loading may be responsible for the easy degradation of the material under both indentations as well as during service operation.

The conventional micro- and macro-Vickers hardness, as well as the standard IIT parameters, suffer from a common weakness when superficial anomalies or RS are present, as in the case of L-PBF fabricated parts. Low hardness values in a tensile RS region can erroneously be interpreted as soft material, and vice versa for compressive RS regions. The complementary use of rate-based properties, such as LSR and H_R_ parameters, can help avoid such a misleading interpretation of hardness values. Additionally, the S_h_-h curve can help elucidate the nature, location, and extension of the RS distribution and other superficial anomalies.

### 4.2. Mechanical Performance of the CZ

At present, the ISE problem and the existence of RS in L-PBF alloys impede any direct correlation between the standard nano-/macro- indentation properties and the microstructure [42]. The LSR and H_R_ introduced here have alleviated such difficulty, as their dependence on ISE has been lowered significantly, except for a small depth range near the origin. In addition, it has been shown that the mean values of LSR and H_R_, as deduced from the multiple-load n-IITs, are consistent with those measured by multi-load M-IITs, within the limits of their standard deviation. Although the n-IITs detected the largest LSR and H_R_ values in the top-right region of the CZ, the M-ITs assigned the largest values to indentation 13, beside the top-right region of the CZ. Such consistency between the n-IITs and M-IITs has enabled us to establish a multiscale correlation of the mechanical performances. Nevertheless, the mechanical performance varied more appreciably in the n-IITs (across the inner grid) than in the M-IITs (along the PLs). This disparity arises because of the bulkier mechanical responses of the material by M-IITs, which probe much larger volumes than n-IITs. The oscillation amplitude in the H_R_ values was larger along the vertical PL than along the horizontal PL, due to the strong and uniform cooling rate effect exerted by the substrate along its length, especially near its adjacent layers. The substrate effect tends to vanish near deposit CZ, as can be seen from the n-IITs, which confirmed a lower mechanical performance at the bottom.

Figure 12 is a preliminary attempt of correlation between H_R_ and the GND density. Although GND density is just a portion of the overall dislocation density and the number of indentations within each region is quite limited, as shown by the large scatter among the measured data, the collected values of H_R_ and GND density clearly identify the subregion R as the one with the most enhanced mechanical performance. A more accurate correlation could be obtained with a more precise measurement of the dislocation density [43]. On the other hand, the number of indentations in subregion R cannot be increased arbitrarily without affecting the surrounding indents’ strain field. A large dislocation density in AM alloys is the principal reason for their outstanding combination of strength and ductility, in comparison to conventional materials [5].

The M-IIT results shown here prove that H_R_ can be considered as a reference (ISE-free) bulk mechanical property that can be related with the strength properties obtained from conventional tensile/compressive tests. It provides a local measurement of the bulk elastoplastic response of the material, which can be viewed as an intricate combination of the microstructure strength and the stable RS that survive until the last stage of indentation loading.

IIT inevitably always leads to the relaxation of as-built RS in L-PBF samples, because of their generated plasticity. Indeed, only unstable RS states can usually be relaxed during indentation. Weak stability may be caused either by the initial fabrication process or by the technique selected for slicing and/or preparing the sample. Susceptibility to RS relaxation depends upon the selected peak load and the sign and distribution of the internal RS. Although n-IIT may relax the RS in the short range (i.e., cell-wall size), M-IITs tend to alter them over the deposit scale in both depth and width, with an increasing peak load. In this study, other M-IIT attempts, carried out days after the two M-IIT PLs, in the narrow gaps within the n-IIT grid, resulted in surprising abnormal ICs, like those of [10], even after carefully fulfilling the standard recommendations on sample preparation, frame compliance correction, and separation distances between indents [23]. This unexpected behavior of the as-built L-PBF deposits clearly suggests that all M-IITs should be performed just after n-IITs, while ensuring a sufficient separation distance between the indents and the edges to minimize compliance problems in the sample.

Although the anisotropy indices measured here are lower than those reported for similar deposits [38], the IPFs of the three subregions (R, C, and B) in the CZ have revealed the presence of some crystal anisotropy, which could be related with the measured anisotropic mechanical performance across the sample. The latter naturally arises from the used fabrication strategy, kinetic (solidification rate) and thermal factors (e.g., thermal gradients, cooling rates) set across the CZ. These, in turn, are influenced by the differential cooling effects exerted by the edges and by the substrate. Surprisingly, some anisotropy effects remained in the CZ, despite it being about 6 mm away from the edges. Specifically, crystal anisotropy predominated in subregion R along the longitudinal (X) and building directions (Z). The latter was caused by the thermal gradient that set in across the deposit, because of the different cooling rates acting on the left and right edges of the deposit. This state can be proved by considering the considerable slope in E_IT_ in Figure 6c, which is the property that is the most sensitive to RS, as compared to the E_IT_ values for the same loads across the build direction. The relatively larger cooling rate at the right edge is consistent with the measured smaller grain area (215 µm^2^), and it is attested by the greater mechanical performance of subregion R than of the other two subregions. Conversely, subregion B exhibits a dominant crystal anisotropy along the transversal, i.e., horizontal direction, parallel to the substrate surface. This is associated with the prevailing cooling effect that is imparted by the substrate to the adjacent layers of the deposit during the deposition process. Despite this, the grain size in B is slightly larger (257 µm^2^) than that in subregion R, due to B being affected by less intense cooling than the corners or edges. This explains why its mechanical response is lower, albeit fair in absolute terms. Conversely, the inner subregion, C, of the CZ shows a weaker and more widespread anisotropy effect. Any stronger anisotropic effects, created by the edges and the substrate, are naturally diminished in the inner subregion of the CZ. Moreover, this subregion is expected to be the last to cool, and the heat is therefore accumulated for a longer time, thereby directly impacting its average grain size (267 µm^2^), which is larger than those in the other two subregions. Nevertheless, its mechanical performance is relatively good, considering its higher GND density.

Finally, LSR enables a successful comparison to be made of the mechanical performance of different 316L deposits undergoing different deposition strategies with different process parameters under different testing peak loads. In [15], the average LSR across the CZ of the deposit (YZ plane) was around 44 GPa, which is considerably lower than the 56 GPa measured in the present study for the n-IITs or the 57.3 GPa measured for the M-IITs. This difference in LSR can be ascribed to a coarser cellular microstructure (>3 µm) in the former versus the submicron size considered in the current study.

## 5. Conclusions

Both the S_h_-h plots and H_R_ (or LSR) are used here to complement the standard indentation properties (H_IT_ and E_IT_) in the determination of the mechanical performance of an L-PBF 316L SS alloy. The S_h_-h plots allow any sort of superficial anomaly (e.g., heterogeneities, microstructure defects, RS, indenter bluntness, etc.) to be analyzed quantitatively. The susceptibility of the initial RS to relax during indentation can be assessed by analyzing the slope changes in the S_h_-h plot.

H_R_ and LSR are rate-dependent, elastoplastic bulk material properties embodying stable short- and eventually long-range RS effects. The proposed multiload/multiscale indentation test methodology becomes very effective when the mechanical performance is demanded at different depths, taking advantage of the ISE-free feature of the H_R_ and LSR. The CZ of a sliced deposit bonded with a (steel) substrate was thoroughly investigated. The following results were achieved:

- the RSD of H_R_ (or LSR) was found to be lower than that of H_IT_ or E_IT_ across all the loads, signifying that the former is less affected by ISE;
- the RSD of all the indentation parameters was maximum for the lowest peak load (50 mN), highlighting the increased surface sensitivity of the measurements at this load in comparison to higher loads;
- the contour maps of the elastoplastic properties (LSR and H_R_) over the deposit core zone (CZ) underlined an enhanced mechanical performance along the transversal direction of the deposit, i.e., from the right to the left edge; this anisotropy effect was confirmed by means of EBSD crystal anisotropy analysis;
- the oscillation period (observed in few M-IITs) of S_h_-h curve in the Y-Z plane was estimated as 20 ± 5 µm, which is equivalent to about a 200 µm radius of the elastoplastic region underneath the indenter (approximately double the hatching distance used during fabrication);
- the oscillation amplitude in the H_R_ values (via M-IIT) was larger along the vertical PL than along the horizontal PL, due to the strong and uniform cooling rate by the substrate along the width of the deposit, especially near its adjacent layers;
- the mechanical performance of the tested L-PBF 316L alloy showed quite consistent indentation modulus average of 201 GPa, slightly higher than the reference Young’s modulus (190 GPa), thus leading to mean values of 2.3 and 2.4 GPa for H_R_ and H_IT_, respectively, at all loads, over the two performance lines;
- the mean value of the H_R_ (2.3 GPa) at all n-IIT loads was consistent with that of the M-IIT, while the H_IT_ and E_IT_ were more affected by ISE.

As per the three subregions (R, C, and B) of the CZ investigated by means of EBSD and n-IITs, the results revealed the following:

- the moderate levels of crystal anisotropy were correlated with the anisotropy of the measured large-scale mechanical performance, and subsequently correlated with the processing and microstructure parameters;
- subregions R and B showed stronger anisotropy effects, due to their vicinity to the (cooling) edges and the substrate, respectively;
- the main reason for large-scale (transversal) anisotropy was ascribed to the strong cooling effect from the substrate, whereas the cooling edges of the deposit turned out to be the second main reason for large-scale anisotropy;
- the inner subregion C of the CZ was the last region to cool down, and it showed a relatively good mechanical performance, despite its large grain size;
- best mechanical performance was found in the subregion R (in compression state) near the right edge of the deposit, which exhibited on average 2.5 GPa, 215 µm^2^, and 1.3 × 10^14^ m^−2^ for H_R_, grain cross section area, and GND density, respectively.

Undesirable sample compliance problems induced by RS relaxation can be prevented by performing n-IITs before the M-IITs.

Future research is prospected towards a more robust experimental validation of the developed multiload/multiscale indentation test against alternative methods enabling the measurement of RS at different depths.

## Figures and Tables

**Table 1:** Chemical composition of the 316L deposit.

Elements	C	Cr	N	Mn	Mo	Ni	P	S	Si	Fe
weight (%)	0.049	16.408	0.108	0.587	2.286	12.345	0.020	0.009	0.499	bal

**Table 2:** L-PBF process parameters [29].

Laser Power	Layer Thickness	Scan Speed	Size Distribution of Raw Powder	Hatch Spacing
300 W	50 μm	1 ms^−1^	15–45 μm	110 μm

**Table 3:** Mean and RSD percentage values of the n-IIT properties for each peak load.

Loads (mN)	LSR		H_R_		H_IT_		E_IT_	
	Mean (GPa)	RSD (%)	Mean (GPa)	RSD (%)	Mean (GPa)	RSD (%)	Mean (GPa)	RSD (%)
50	58.2	13	2.38	13	3.30	14	207	12
100	56.3	8.0	2.30	8.0	3.03	8.0	210	6.6
150	56.0	6.9	2.29	6.9	2.90	6.8	220	9.9
200	54.7	7.5	2.24	7.5	2.75	9.3	237	9.8
All Loads	56.3	9.0	2.30	9.0	3.00	12	219	11

**Table 4:** Mean and RSD values of the M-IIT properties for each peak load. The considered H_IT_ and E_IT_ values were obtained after the fourth and the last loading cycle for each indentation.

Loads (N)	LSR		H_R_		H_IT_		E_IT_	
	Mean (GPa)	RSD (%)	Mean (GPa)	RSD (%)	Mean (GPa)	RSD (%)	Mean (GPa)	RSD (%)
50	56.9	1.5	2.32	1.5	2.33	8.3	203	4.5
100	58.4	2.1	2.38	2.1	2.49	2.3	205	3.6
150	56.6	3.6	2.31	3.6	2.42	1.8	198	1.1
200	56.9	1.0	2.32	1.0	2.41	2.3	197	3.6
All Loads	57.3	2.5	2.33	2.5	2.41	5.3	201	3.9

## Data Availability

The original contributions presented in this study are included in the article. Further inquiries can be directed to the corresponding author.
